# Barbaric Dentistry

**Published:** 1899-10

**Authors:** 


Barbaric Dentistry.—In America the fad of showing a gold crown in the front of the mouth seems on the increase, and gold crowns are also advertised for vulcanizing on to artificial dentures. A writer to the New York *Journal* thus bewails the barbarous and inartistic custom: "Allow me to call your attention to the gold tooth which is becoming so popular with the young ladies of this country. I am desirous of interesting you in this subject, so you will by your influence advise the people, through the New York *Herald*, to stop having one of the greatest gifts they possess destroyed (their teeth) just to have one of those unsightly gold teeth placed in the front of their mouths. What a pity it is that the American girls indulge in such a barbarous custom! It is not unlike one of the characteristic traits of some of the tribes on the Philippine Islands, who blacken their teeth to make themselves look more beautiful." So far, our young ladies seem to have more sense.

